# A Laboratory Course in Bacteriology

**Published:** 1902-09

**Authors:** 


A LABORATORY COURSE IN BACTERIOLOGY. For the Use of Medical, Agricultural and Industrial Students. By FREDERIC P. GORHAM, A. M., Associate Professor of Biology, Brown University; Bacteriologist, Health Department, Providence, R. I. 182 pages, with 97 illustrations. Philadelphia and London: W. B. Saunders & Company. 1901. [Price, $1.25 net.]

This book intended only as a laboratory guide in bacteriology for medical, agricultural, and industrial students, is all that could be expected of it, considering the small number of pages it contains. While it is not designed exclusively for the student of any particular branch, it is a very practical preparatory guide from which he may comprehensively continue the study upon the special lines he selects.

The author has the subject-matter well in hand, as is shown by the admirable arrangement of his topics; his clear and concise directions, as to procedures in technique; the staining methods, formulæ, and the like, are reliable and effective.

A few unimportant omissions and errors are found, for instance: Fig. 88, representing tubercle bacilli in sputum should have been credited to the source from which it is copied, as has been properly done with various other illustrations. (It was first known to us in the Compend on Bacteriology, by M. V. Ball.) There is an error in the references to figs. 16 and 87. Fig. 89, copied from Frankel and Pfeiffer represents a pure culture of tubercle bacilli, not tubercle bacilli in sputum, also some of the reproductions of bacteria, as figs. 65 and 77, are poorly done. Stress should have been laid on the diagrammatic character of the illustration of the Widal reaction (fig. 85).

